# Mental Health Effects in Primary Care Patients 18 Months After a Major Wildfire in Fort McMurray: Risk Increased by Social Demographic Issues, Clinical Antecedents, and Degree of Fire Exposure

**DOI:** 10.3389/fpsyt.2019.00683

**Published:** 2019-09-18

**Authors:** Shahram Moosavi, Bernard Nwaka, Idowu Akinjise, Sandra E. Corbett, Pierre Chue, Andrew J. Greenshaw, Peter H. Silverstone, Xin-Min Li, Vincent I. O. Agyapong

**Affiliations:** ^1^Department of Psychiatry, Faculty of Medicine and Dentistry, University of Alberta, Edmonton, AB, Canada; ^2^Department of Family Medicine, Faculty of Medicine and Dentistry, University of Alberta, Edmonton, AB, Canada; ^3^Department of Psychiatry, Northern Lights Regional Health Centre, Fort McMurray, AB, Canada

**Keywords:** wildfire, major depressive disorder, generalized anxiety disorder, mental health, anxiety disorder, support, counseling

## Abstract

**Objectives:** To assess prevalence of *likely* posttraumatic stress disorder (PTSD), major depressive disorder (MDD), and generalized anxiety disorder (GAD) in patients attending the only out-of-hours primary care clinic in Fort McMurray some 18 months following a major fire.

**Methods:** A quantitative cross-sectional survey was used to collect data through self-administered paper-based questionnaires to determine *likely* PTSD, MDD, and GAD using the PTSD Checklists for Diagnostic and Statistical Manual (DSM) 5, Patient Health Questionnaire (PHQ) 9, and GAD-7, respectively, from residents of Fort McMurray who were impacted by the wildfires. This was carried out eighteen (18) months after a major wildfire, which required the rapid evacuation of the entire city population (approximately 90,000 individuals).

**Results:** We achieved a response rate of 48% and results from the 290 respondents showed the 1 month prevalence rate for likely PTSD was 13.6%, likely MDD was 24.8%, and likely GAD was 18.0%. Compared to self-reported prevalence rates before the wildfire (0%, 15.2%, and 14.5% respectively), these were increased for all diagnoses. After controlling for other factors in a logistic regression model, there were statistically significant associations between individuals who had likely PTSD, MDD, and GAD diagnoses and multiple socio-demographic, clinical, and exposure-related variables as follows: PTSD: History of anxiety disorder and received counselling had odds ratios (ORs) of 5.80 and 7.14, respectively. MDD: Age, witnessed the burning of homes, history of depressive disorder, and receiving low level support from friends and family had ORs of 2.08, 2.29, 4.63, and 2.5, respectively. GAD: Fearful for their lives or the lives of friends/family, history of depressive disorder, and history of anxiety disorder had ORs of 3.52, 3.04, and 2.68, respectively. There were also associations between individuals with a likely psychiatric diagnosis and those who also had likely alcohol or drug abuse/dependence.

**Conclusion:** Our study suggests there are high prevalence rates for mental health and addiction conditions in patients attending the out-of-hours clinic 18 months after the wildfires, with significant associations between multiple variables and likely PTSD, MDD, and GAD. Further studies are needed to explore the impact of population-based mental health interventions on the long-term mental health effects of the wildfires.

## Introduction

The Fort McMurray wildfire of 2016 ranks as the most expensive in Canadian history, burned 5,890 km^2^ of land, and destroyed more than 2,400 buildings (1). The severe wildfire forced almost 90,000 residents out of the Regional Municipality of Wood Buffalo in a rapid, and unplanned, exodus (2) when a mandatory evacuation order was issued on the 3^rd^ of May 2016, which remained in place until the 1^st^ of June 2016. The major fire was nicknamed “The Beast,” and the Fort McMurray wildfire changed the landscape—and tens of thousands of lives—forever (3). Mental health issues especially depression and anxiety are the primary cause of disability in Canada (4). By the age of 40, half of Canadians already have or have had at least one psychiatric problem that can be the cause for other numerous issues (4). In this group, the likelihood of substance use disorder is doubled, and mean survival lengths are decreased by 10–20 years compared to those who do not have mental health problems in their community (5–7). These impacts also have major economic implications, with these conditions costing Canada around $51 billion annually when considering both direct and indirect costs (health care cost, lost working hours, and lower productivity) (4, 8). There is compelling evidence demonstrating that addressing these mental health issues has high economic returns, and not just a reduction in morbidity (9, 10). The impact of natural disasters on the mental health of victims has been researched over the past two decades (11), with studies examining mental health impacts on survivors of disasters like Hurricane Katrina in 2005, Hurricane Sandy in 2012, the Fukushima nuclear disaster in 2011, and the terror attack on the World Trade Center in 2001. Extensive findings from these, and other natural disasters, suggest that a proportion of survivors will continue to have mental health issues for an extended period of time following the disaster, and sometimes for the rest of their lives (11–28).

The most studied psychiatric pathologies after disasters are posttraumatic stress disorder (PTSD) and major depressive disorder (MDD) (11, 14, 23, 25), with generalized anxiety disorder (GAD) (11) being less frequently studied. Similarly, problematic drug and alcohol use and nicotine dependence and their relationship with other mental health disorders have also not been extensively studied previously (11, 13, 14).

Many socio-demographic and clinical factors have been documented to predict which individuals are likely to suffer from the longer-term mental health impacts of disasters. These include individual resilience, healthy or maladaptive coping skills, the severity of the disaster, degree of involvement of the victim, preexisting mental health issues, gender, age, social support and relationships, support from the government, support from insurance companies, and endurance of the mental health issue (11, 29–32). However, there have been no previous studies that have examined longer-term mental health impacts of a wildfire as seen in a primary care setting. The purpose of this study is therefore to understand the mental health impacts of the Fort McMurray wildfire on residents seeking medical care in the cities only out-of-hours primary care center, some 18 months after the wildfire. In addition, we aim to further identify other potential predictive factors for subsequent development of wildfire-related mental health conditions including socio-demographic, clinical, and wildfire-exposure factors. We hypothesized that as a result of the wildfires, the prevalence rates of the mental health disorders being studied would be higher than previously recorded prevalence rates for these disorders within the entire population. We also hypothesize that predictive factors for these conditions would be similar to those previously recognized for post-natural disaster populations. Additional insights gained from this information may support future health planning for mental health interventions at the primary care level post-disaster.

## Materials and Methods

### Study Setting

Fort McMurray is the urban service area of the Regional Municipality of Wood Buffalo in Northern Alberta with a city population of 82,724 in 2015 (33), but there are an additional 30,000 individuals who were working in camps linked to the city. Fort McMurray has only a single out-of-hours family practice medical clinic. This is operated by the Wood Buffalo Primary Care Network (PCN) between 5pm and 10pm from Monday to Friday, and 11am till 8pm at weekends. In May 2016, a raging wildfire threatened life and property and destroyed about 2,400 homes in Fort McMurray and surrounding communities.

### Study Design, Sample Size, and Institutional Review Board Approval

Quantitative data collected through self-administered paper-based questionnaires were used in this cross-sectional survey study design. An anticipated sample size of 460 was determined based upon a 95% confidence interval; the sample size needed to estimate the prevalence rates for mental disorders with a margin of error of ±4.0% will be 455. This was considered very feasible as this research team achieved a similar sample size for a related study in Fort McMurray at 6 months post-wildfire, which targeted 1,500 residents (34–36) and the average monthly adult residents accessing the PCN clinic in 2017 was 1,873. Based on this sample size projection and an expected dropout/incomplete data rate of about 30% (34–36), random sampling procedures were used to distribute survey questionnaires to 600 adult residents attending the PCN clinic in November 2017. This study was carried out in accordance with the recommendations of the University of Alberta Review and Ethics Board. All subjects gave written informed consent in accordance with the Declaration of Helsinki. The protocol was approved by the University of Alberta Review and Ethics Board (Pro00066054).

### Eligibility, Data Collection, and Analysis

Residents of Fort McMurray who were 18 years of age or older and were able to read and provide written informed consent and were accessing medical services at the out-of-hours PCN clinic in Fort McMurray during the month of November 2017 were invited to participate in the study. A research coordinator approached 600 adult patients registering at the reception area to receive services at the out-of-hours clinic on a sequential basis, and offered them information leaflets about the study. After providing informed consent, the respondents completed paper-based surveys at the clinic. The questionnaires generally took 15–20 min to complete and no incentives were offered to the respondents. Respondents’ demographic and clinical as well as wildfire exposure and support-related information were collected with a data collection form designed for the purpose (34–36).

The PTSD Checklists for Diagnostic and Statistical Manual (DSM) 5, Patient Health Questionnaire (PHQ) 9, and GAD-7 were used to assess the presence or absence of likely PTSD, MDD, and GAD, respectively, in respondents. The PTSD Checklist for DSM 5 (PCL 5) Part 3 (37) was used to assess *likely PTSD* in respondents. Patients with a PCL-5 score of 33 or more were deemed to have a *likely* PTSD. The PHQ-9 scoring was done using the standard recommendation with threshold for likely depression being met if 5 of the 9 items were checked at least “more than half the days” and either item A or B was checked at least “more than half the days” (38). A score of 10 or more on GAD-7 was used to assess GAD symptomatology (39). The Alcohol Use Disorder Identification Test (AUDIT) (40) was used to assess the presence of problem drinking and the Drug Use Disorder Identification Test (DUDIT) (41) was used to assess for the presence of drug-related problems among the respondents.

We analyzed data using SPSS Version 20 (42). We presented absolute numbers and percentages according to gender for all the demographic and clinical variables. We used univariate analyses with chi-square tests to ascertain the relationship between each of the predictors and the likelihood that respondents had PTSD, MDD, or GAD. Variables with a statistically significant relationship (p ≤ 0.05, two-tailed exact significance) with the likelihood of PTSD, MDD, or GAD, respectively, on univariate analysis, together with predictors that had trends towards significant (0.05 ≤ p ≤ 0.1, two-tailed exact significance), were then entered into a logistic regression model.

Correlation diagnostics were performed before performing the logistic regression analysis to ensure that very strong correlations (Spearman’s correlation coefficient of 0.7 to 1.0 or −0.7 to −1.0 on correlation diagnostics) (43) among predictor variables were avoided. Consequently, variables including: “sought counseling after the wildfire,” “had no history of mental health diagnosis before the wildfire,” and “was on no psychotropic medication before the wildfire,” which were respectively highly correlated with “received counseling after the wildfire,” “had a history of depressive disorder,” and “was on an antidepressant,” were dropped from the regression model. Odds ratios (ORs) from the binary logistic regression analysis were examined to determine the association between each of the variables in the model and the likelihood of respondents presenting with likely PTSD, MDD, or GAD, controlling for the other variables in the model. For each categorical variable in the logistic regression model, the first category was used as the base for comparison.

## Results

Of the 600 survey forms distributed to patients attending a primary care out-of-hours clinic in Fort McMurray in the month of November 2017, a total of 290 completed questionnaires were returned giving a response rate of 48.0%. With the lower sample size of 290 rather than the projected 460, the margin of error for our prevalence rate estimates was ±5.3%.

### Descriptive Sample Characteristics

**Table 1** provides descriptive summaries of the demographic and clinical correlates of the respondents.

**Table 1:** Descriptive Characteristics of the Sample.

Variable Type	Variables	Overall N (%)
**Sociodemographic**	**Age (Years)** ≤40 ≥41	158 (54.5%) 130 (44.8%)
	**Gender** Male Female	130 (45.1%) 158 (54.9%)
	**Employment status** Employed Unemployed	230 (79.6%) 59 (20.4%)
	**Relationship status** Married/cohabiting/partnered Single/separated/divorced/widowed	204 (72.1%) 79 (27.9%)
**Living situation**	**Where respondents lived after the wildfires relative to where they lived before the wildfire** Same home they lived in before the fire Different home although previous home was not destroyed by the fire Different home because previous home was destroyed by the fire	214 (74.6%) 48 (16.7%) 25 (8.7%)
**Exposure to disaster**	**Area of residence relative to destroyed properties** 0–1.0 properties destroyed per kilometer square 1.1–50.0 properties destroyed per kilometer square 50.1–300.0 properties destroyed per kilometer square	79 (28.3%) 173 (62%) 27 (9.7%)
	**Respondents witnessed burning of homes by the wildfires** Yes No	190 (67.1%) 93 (32.9%)
	**Respondents were fearful for their lives or the lives of friends/family** Yes No	223 (78.8%) 60 (21.2%)
	**How frequently did respondents watch television images about the devastation caused by the wildfires during the period of the evacuation** Daily Less frequently than daily	235 (82.2%) 51 (17.8%)
	**How frequently did respondents read newspaper and internet articles related to the devastation caused by the wildfires** Daily Less frequently than daily	231 (79.9%) 59 (20.3)
	**Home was completely destroyed by the wildfires** Yes No	31 (10.8%) 257 (89.2%)
**Clinical history**	**Respondent had a history of depressive disorder before the wildfires** Yes No	44 (15.2%) 245 (84.4%)
	**Respondent had a history of anxiety disorder before the wildfires** Yes No	42 (14.5%) 247 (85.5%)
	**Respondent had no history of mental health diagnosis before the wildfires** Yes No	66 (22.8%) 223 (77.2%)
	**Respondents were on antidepressants before the wildfires** Yes No	44 (15.2%) 245 (84.6%)
	**Respondents were on no psychotropic medication before the wildfires** Yes No	51 (17.8%) 235 (82.2%)
**Support**	**Received sufficient support from family and friends** High level support Limited or no support	188 (65.3%) 100 (43.7%)
	**Received sufficient support from the Red Cross** High level support Limited or no support	144 (50.7%) 140 (49.3%)
	**Received sufficient support from the government** High level support Limited or no support	98 (34.8%) 184 (65.2%)
**Post-crisis counseling**	**Sought counseling after the wildfires** Yes No	47 (16.4%) 240 (83.6%)
	**Received counseling after the wildfires** Yes No	41 (14.2%) 248 (85.8%)
**Subjective measures**	**Self-reported increased alcohol use after the wildfires** Yes No	34 (11.8%) 254 (88.2%)
	**Self-reported increased drug use after the wildfires** Yes No	12 (4.2%) 277 (95.8%)
**Objective measures**	**Respondents had elevated symptoms consistent with MDD** (based on PHQ-9 scale) MDD likely No MDD	71 (24.8%) 215 (75.2%)
	**Respondents had elevated symptoms consistent with GAD** (based on GAD-7 scale) GAD likely No GAD	51 (18.0%) 232 (82.0%)
	**Respondents had elevated symptoms consistent with PTSD** (based on PTSD Checklist for DSM 5 Part 3 scale) PTSD likely No PTSD	37 (13.6%) 236 (84.6%)
	**Alcohol Use Identification Test (AUDIT)** ≤7 (low risk drinking or abstinence) ≥8 (high risk, harmful, or hazardous drinking or alcohol dependence)	221 (85%) 39 (15%)
	**Drug Use Identification Test (DUDIT)** ≤5 for men and ≤1 for women (no drug-related problems) ≥6 for men and ≥2 for women (drug-related problems)	260 (90.9%) 26 (9.1%)

**Table 1** shows that prior to the onset of the wildfires, 0%, 15.2%, 14.5% of respondents had self-reported histories of PTSD, MDD, and GAD, respectively. In contrast, the results found that 18 months after the wildfires, the 1 month prevalence rate for likely PTSD was 13.6%, for likely MDD was 24.8%, and for likely GAD was 18.0%. Thus, the 1 month prevalence rates of likely PTSD, MDD, and GAD were higher 18 months after the wild fires in this population than life-time permanence rates for these disorders before the wildfires. **Table 1** also indicates that the 1 month prevalence rate for likely problematic/hazardous drinking and drug use was high at 15% and 9.1%, respectively.

### Associations Between Sociodemographic, Clinical, Exposure-Related, and Support Variables and Elevated PTSD, MDD, and GAD Symptoms

**Table 2** illustrates statistically significant associations between 10 socio-demographic and clinical variables including: having a history of a depressive or anxiety disorder, or having no prior mental health diagnosis before the wildfire, being on an antidepressant or on no psychotropic medication before the wildfire, receiving limited or no support from the Red Cross or the Government of Alberta after the wildfire and also seeking and receiving counseling after the wildfires, and the likelihood of having a PTSD 18 months after the wildfires. Similarly, **Table 2** illustrates statistically significant associations between 14 socio-demographic and clinical variables, namely: age, employment status, witnessing the burning of homes, being fearful for their lives and/or those of their loved ones’, having a history of a depressive or anxiety disorder, or having no prior mental health diagnosis before the wildfires, being on an antidepressant or on no psychotropic medication before the wildfire, receiving limited or no support from family/friends, the Red Cross, or the Government of Alberta after the wildfires and also seeking and receiving counseling after the wildfires, and the likelihood of having an MDD 18 months after the wildfires. Finally, **Table 2** illustrates statistically significant associations between 12 socio-demographic and clinical variables, namely: employment status, witnessing the burning of homes, being fearful for their lives and/or those of their loved ones’, having a history of a depressive or anxiety disorder, or having no prior mental health diagnosis before the wildfires, being on an antidepressant or on no psychotropic medication before the wildfires, receiving limited or no support from family/friends or the Red Cross after the wildfires and also seeking and receiving counseling after the wildfires, and the likelihood of having a GAD 18 months after the wildfires.

**Table 2:** Chi-square test of association between demographic and clinical antecedents and the likelihood that the respondents had PTSD, MDD, and GAD.

Variables	Post-traumatic stress disorder			Major depressive disorder			Generalized Anxiety Disorder		
	PTSD likely	P- value	Effect size (Phi/cramer’s V^*^)	MDD likely	P-value	Effect size (Phi/cramer’s V^*^)	GAD likely	P- value	Effect size (Phi/cramer’s V^*^)
**Sex** Male Female	15(12.5%) 22(14.6%)	0.62	0.03	33 (25.8%) 38 (24.4%)	0.78	-0.02	18(14.4%) 33(21.2%)	0.14	0.09
**Age (Years)** ≤40 ≥41	27(18.0%) 10(8.30%)	0.02	-0.14	47 (29.9%) 24 (18.9%)	0.03	-0.13	30(19.5%) 21(16.5%)	0.52	-0.04
**Relationship status** Single/Separated/Divorced/Widowed Married/cohabiting/partnered	13(17.8%) 24(12.3%)	0.25	-0.07	21(26.6%) 48(23.9%)	0.64	-0.03	15(19.0%) 35(17.7%)	0.80	-0.02
**Employment status** Employed Unemployed	28(12.9%) 9(16.4%)	0.50	0.04	50(21.9%) 21(36.8%)	0.02	0.14	35(15.6%) 16(28.1%)	0.03	0.13
**Where respondents lived after the wildfires relative to where they lived before the wildfires** Same home they lived in before the fire Different home although previous home was not destroyed by the fire Different home because previous home was destroyed by the fire	27(13.3%) 6(14.0%) 3(12.5%)	0.99	0.01	45(21.4%) 13(27.1%) 10(40.0%)	0.11	0.13	33(15.9%) 10(21.3%) 7(28.0%)	0.26	0.10
**Area of residence relative to destroyed properties** 0–1.0 properties per km^2^ 1.1–50.0 properties per km^2^ 50.1–300.0 properties per km^2^	11(14.9%) 24(14.7%) 2(7.4%)	0.58	0.06	21(26.9%) 40(23.5%) 8(29.6%)	0.72	0.05	9(11.7%) 34(20.2%) 7(25.9%)	0.16	0.12
**Respondents witnessed burning of homes by the wildfires** No Yes	7(9.9%) 29(16.4%)	0.06	-0.12	11(11.8%) 57(30.6%)	0.001	-0.21	9(9.9%) 39(21.1%)	0.02	-0.14
**Respondents were fearful for their lives or the lives of friends/family** No Yes	4(6.9%) 32(15.4%)	0.10	-0.10	6(10.0%) 61(27.9%)	0.004	-0.17	3(5.0%) 45(20.8%)	0.004	-0.17
**Home was completely destroyed by the wildfire** No Yes	34(14.1%) 3(10.0%)	0.54	-0.04	59(23.3%) 11(35.5%)	0.14	0.09	44(17.6%) 7(22.6%)	0.50	0.04
**How frequently did respondents watch television images about the devastation caused by the wildfires during the period of the evacuation** Less frequently than daily Daily	6(12.5%) 31(14.0%)	0.78	-0.02	11(22.0%) 58(25.0%)	0.65	-0.03	7(14.0%) 43(18.8%)	0.43	-0.05
**How frequently did respondents read newspaper and internet articles related to the devastation caused by the wildfires** Less frequently than daily Daily	6(11.3%) 31(14.1%)	0.60	-0.03	18(30.5%) 53(23.3%)	0.26	0.07	9(15.8%) 42(18.6%)	0.62	-0.03
**Respondent had a history of depressive disorder before the wildfires** No Yes	21(9.1%) 16(38.1%)	0.00	0.31	44(18.3%) 27(61.4%)	0.00	0.36	30(12.6%) 21(48.8%)	0.00	0.34
**Respondent had a history of anxiety disorder before the wildfires** No Yes	20(8.6%) 17(41.5%)	0.00	0.34	48(19.8%) 23(54.8%)	0.00	0.29	32(13.3%) 19(46.3%)	0.00	0.30
**Respondent had no history of mental health diagnosis before the wildfires** No Yes	16(7.7%) 21(32.8%)	0.000	0.311	36(16.4%) 35(53.0%)	0.000	0.357	24(11.1%) 27(41.5%)	0.000	0.333
**Respondents were on antidepressants before the wildfires** No Yes	23(10.0%) 13(34.2%)	0.000	0.248	48(19.8%) 21(52.5%)	0.000	0.265	34(14.2%) 16(41.0%)	0.000	0.243
**Respondents were on no psychotropic medication before the wildfires** No Yes	22(10.0%) 14(28.6%)	0.001	0.211	42(18.2%) 27(52.9%)	0.000	0.311	31(13.5%) 19(38.0%)	0.000	0.245
**Received sufficient support from family and friends** High level support Limited or no support	20(11.2%) 17(18.3%)	0.109	0.097	35(18.8%) 36(36.7%)	0.001	0.197	27(14.7%) 24(24.7%)	0.037	0.124
**Received sufficient support from the Red Cross** High level support Limited or no support	9(6.5%) 27(20.8%)	0.001	0.210	23(16.1%) 45(32.6%)	0.001	0.193	18(12.9%) 32(23.2%)	0.025	0.134
**Received sufficient support from the Government of Alberta** High level support Limited or no support	6(6.4%) 29(16.9%)	0.016	0.148	16(16.7%) 51(28.0%)	0.035	0.126	12(12.6%) 36(20.0%)	0.126	0.092
**Sought counseling after the wildfires** No Yes	16(7.1%) 21(45.7%)	0.000	-0.421	48(20.3%) 23(48.9%)	0.000	-0.245	31(13.3%) 20(42.6%)	0.000	-0.283
**Received counseling after the wildfires** No Yes	18(7.8%) 19(46.3%)	0.000	-0.403	50(20.4%) 21(52.5%)	0.000	-0.258	34(14.0%) 17(42.5%)	0.000	-0.258

### Predictors of Elevated PTSD Symptoms

Seven of the predictor variables for likely PTSD in **Table 2** with significant p-values (p ≤ 0.5) and those with near significant p-values (0.5 < p ≤ 0.1) were entered into the logistic regression model. Three variables that were highly correlated with other included variables were thus excluded from the model. The full model containing all eight predictors was statistically significant, *X* ^2^ (7, N = 258) = 61.70, p < 0.00, indicating that the model was able to distinguish between respondents who had likely PTSD at 18 months versus those who did not. The model as a whole explained between 21.3% (Cox and Snell R^2^) and 38.8% (Nagelkerke R^2^) of the variance in the likelihood that respondents will present with likely PTSD and correctly classified 86.4% of all cases.

As shown in **Table 3**, only two of the independent variables (age, witnessed burning of homes, history of anxiety disorder, and received counseling after the wildfires) made unique statistically significant contributions to the model. The OR for “history of anxiety disorder” was 5.80, which suggests that respondents who had histories of anxiety disorder and older were about six times more likely to present with likely PTSD 18 months after the wildfires, controlling for all other factors, compared to respondents who did not have histories of anxiety disorder. Similarly, respondents who received counseling after the wildfires were about seven times more likely to present with likely PTSD at 18 months compared with respondents who reported they did not receive counseling when all the other factors are controlled for.

**Table 3:** Predictors of PTSD symptomatology.

Predictor	B	SE	Wald	df	P-value	Odds Ratio	95% CI for odds ratio	
							Lower	Upper
**Age (Years)** ≤40 ≥41	-0.52	0.46	1.24	1	0.27	0.60	0.24	1.48
**Respondents witnessed burning of homes by the wildfires** No Yes	0.24	0.55	0.19	1	0.67	1.27	0.43	3.71
**Respondent had a history of depressive disorder before the wildfires** No Yes	0.55	0.58	0.90	1	0.34	1.73	0.56	5.39
**Respondent had a history of anxiety disorder before the wildfires** No Yes	1.76	0.56	9.71	1	0.002	5.80	1.92	17.50
**Received sufficient support from the Red Cross** High level support Limited or no support	0.75	0.65	1.36	1	0.24	2.13	0.60	7.55
**Received sufficient support from the Government of Alberta** High level support Limited or no support	0.73	0.75	0.96	1	0.33	2.84	0.48	9.07
**Received counselling after the wildfires** No Yes	1.97	0.48	16.86	1	0.00	7.17	2.80	18.37
**Constant**	-3.93	0.70	31.412	1	0.000	0.020		

### Predictors of Elevated MMD Symptoms

Ten of the predictor variables for likely MDD in **Table 2** with significant p-values (p ≤ 0.5) and those with near significant p-values (0.5 < p ≤ 0.1) were entered into the logistic regression model. Four variables that were highly correlated with other included variables were thus excluded from the model. The full model containing all 10 predictors was statistically significant, *X* ^2^ (10, N = 267) = 65.11, p < 0.00, indicating that the model was able to distinguish between respondents who had likely MDD at 18 months versus those who did not. The model as a whole explained between 21.6% (Cox and Snell R^2^) and 32.4% (Nagelkerke R^2^) of the variance in the likelihood that respondents will present with likely MDD and correctly classified 78.3% of all cases.

As shown in **Table 4**, only four of the independent variables (age, witnessed burning of homes, history of depressive disorder, and support from family/friends) made unique statistically significant contributions to the model. The OR for “age” was 2.08, 95% CI of 1.3–20.1, which suggests that respondents who were aged 41 and older were about two times more likely to present with likely MDD 18 months after the wildfires, controlling for all other factors, compared to respondents who were 40 years or younger. Similarly, respondents who witnessed the burning of homes and those with a history of depressive disorder were about two and four and a half times, respectively, more likely to present with likely MDD at 18 months compared with respondents who reported they did not witness the burning of homes and those without histories of depressive disorders when all the other factors are controlled for. Finally, respondents who reported they had only low level support from friends and family were about two and a half times more likely to present with likely MDD at 18 months compared with respondents who reported they received absolute support from friends and family when all the other factors in the model are controlled for.

**Table 4:** Predictors of MDD symptomatology.

Predictor	B	SE	Wald	df	P-value	Odds Ratio	95% CI for Odds Ratio	
							Lower	Upper
**Age (Years)** ≤40 ≥41	-0.73	0.36	4.25	1	0.04	2.08	1.04	4.17
**Employment status** Employed Unemployed	0.54	0.41	1.78	1	0.18	1.72	0.77	3.83
**Respondents witnessed burning of homes by the wildfires** No Yes	0.83	0.42	3.96	1	0.05	2.29	1.01	5.19
**Respondents were fearful for their lives or the lives of friends/family** No Yes	0.86	0.51	2.87	1	0.09	2.37	0.87	6.41
**Respondent had a history of depressive disorder before the wildfire** No Yes	1.53	0.49	9.74	1	0.00	4.63	1.77	12.12
**Respondent had a history of anxiety disorder before the wildfire** No Yes	0.25	0.52	0.23	1	0.63	1.28	0.47	3.53
**Received sufficient support from family and friends** High level support Limited or no support	0.89	0.37	5.90	1	0.02	2.43	1.19	4.97
**Received sufficient support from the Red Cross** High level support Limited or no support	0.540	0.45	1.42	1	0.23	1.72	0.71	4.17
**Received sufficient support from the Government of Alberta** High level support Limited or no support	-0.06	0.51	0.01	1	0.91	0.94	0.35	2.57
**Received counselling after the wildfires** No Yes	0.785	0.44	3.24	1	0.07	2.19	0.93	5.16
**Constant**	-3.433	0.61	31.38	1	0.00	0.03		

### Predictors of Elevated GAD Symptoms

Eight of the predictor variables for likely GAD in **Table 2** with significant p-values (p ≤ 0.5) and those with near significant p-values (0.5 < p ≤ 0.1) were entered into the logistic regression model. Four variables that were highly correlated with other included variables were thus excluded from the model. The full model containing all eight predictors was statistically significant, *X* ^2^ (8, N = 269) = 48.86, p < 0.00, indicating that the model was able to distinguish between respondents who had likely GAD at 18 months versus those who did not. The model as a whole explained between 16.6% (Cox and Snell R^2^) and 27.2% (Nagelkerke R^2^) of the variance in the likelihood that respondents will present with likely GAD and correctly classified 82.2% of all cases.

As shown in **Table 5**, only three of the independent variables (fearful for their lives or the lives of friends/family, history of depressive disorder, and history of anxiety disorder) made unique statistically significant contributions to the model. The OR for “fearful for their lives or the lives of friends/family” was 3.52, which suggests that respondents who were fearful for their lives or the lives of friends/family were about three and a half times more likely to present with likely GAD 18 months after the fire, controlling for all other factors, compared to respondents who reported they were not fearful for their lives or the lives of friends/family. Similarly, respondents with a history of depressive disorder or an anxiety disorder were about three and two and a half times, respectively, more likely to present with likely GAD at 18 months compared with respondents without histories of depressive disorders and anxiety disorders when all the other factors are controlled for.

**Table 5:** Predictors of GAD symptomatology.

Predictor	B	SE	Wald	df	P-value	Odds Ratio	95% CI for Odds Ratio	
							Lower	Upper
**Employment status** Employed Unemployed	0.37	0.43	0.74	1	0.39	1.45	0.62	3.40
**Respondents witnessed burning of homes by the wildfires** No Yes	0.37	0.44	0.71	1	0.40	1.45	0.61	3.44
**Respondents were fearful for their lives or the lives of friends/family** No Yes	1.26	0.66	3.70	1	0.05	3.52	0.98	12.71
**Respondent had a history of depressive disorder before the wildfires** No Yes	1.11	0.47	5.62	1	0.02	3.04	1.21	7.61
**Respondent had a history of anxiety disorder before the wildfires** No Yes	0.98	0.48	4.16	1	0.04	2.68	1.04	6.89
**Received sufficient support from family and friends** High level support Limited or no support	0.43	0.39	1.22	1	0.27	1.53	0.72	3.28
**Received sufficient support from the Red Cross** High level support Limited or no support	0.46	0.39	1.35	1	0.25	1.58	0.73	3.40
**Received counseling after the wildfires** No Yes	0.85	0.45	3.60	1	0.06	2.34	0.97	5.61
**Constant**	-4.03	0.70	32.65	1	0.00	0.02		

### Associations Between PTSD, MDD, and GAD Symptoms and Problematic Drug or Alcohol Use

**Table 6** shows associations between self-reported increase in both alcohol and drug use and likely PTSD and MDD but not GAD. In contrast, there was an association between likely drug-related problems but not likely alcohol use disorder as measured with the DUDIT and AUDIT respectively, and likely PTSD, MDD, and GAD.

**Table 6:** Chi-square test of association between likely PTSD, MDD, and GAD and likely abuse/dependence on alcohol and substances.

Variables	Post-traumatic stress disorder			Major depressive disorder			Generalized anxiety disorder		
	PTSD likely	PTSD unlikely	P- value	MDD likely	MDD unlikely	P- value	GAD likely	GAD unlikely	P- value
**Self-reported increased alcohol use after the wildfires** No Yes	28(77.77%) 8(22.22%)	210(89.36%) 25(10.63%)	0.05	56(80%) 14 (20%)	194(90.65%) 20(9.34%)	0.02	42(84%) 8(16%)	205(88.74%) 26(11.25%)	0.35
**Self-reported increased drug use after the wildfires** No Yes	30(83.33%) 6(16.66%)	230 (97.45%) 6(2.54%)	0.00	64 (91.42%) 6 (8.57%)	209(97.20%) 6(2.79%)	0.04	46(92%) 4(8%)	224(96.55%) 8(3.44%)	0.15
**Alcohol use identification test scores** ≤7 (low risk drinking or abstinence) ≥8 (High risk, harmful or hazardous drinking or alcohol dependence)	27(77.14%) 8(22.85%)	191(86.03%) 31(13.96%)	0.17	55(83.33%) 11(16.66%)	164(85.41%) 28(14.58%)	0.68	41(87.23%) 6(12.76%)	178(84.36%) 33(15.63%)	0.62
**Drug use identification test** ≤5 for men and ≤1 for women (no drug-related problems) ≥6 for men and ≥2 for women (drug-related problems)	28 (75.67%) 9 (24.32%)	215 (92.67%) 17 (7.32%)	0.004	54 (78.26%) 15 (21.73%)	202 (94.83%) 11 (5.16%)	0.00	40 (78.43%) 11 (21.56%)	213 (93.42%) 15 (6.57%)	0.002

## Discussion

This study showed an elevated 1 month prevalence rates of the likely PTSD, MDD, and GAD 18 months after the Fort McMurray 2016 wildfire compared to the self-reported rates of PTSD, MDD, and anxiety before the fire. Prevalence rates for PTSD and MDD, but not GAD, were also higher than the rates of these disorders in the entire population 6 months after the wildfire (34–36). Before the disaster, self-reported PTSD, MDD, and anxiety disorder in the respondents were 0%, 15.2%, and 14.5%, respectively. After the fire, 1 month prevalence of likely PTSD, MDD, and GAD increased to 13.6%, 24.8%, and 18.0%, respectively. Interestingly these rates are greater for both likely PTSD and likely MDD when compared to prevalence rates of 12.8% and 14.8% in the Fort McMurray general population 6 months after the wildfire (34–36). This demonstrates that much of the psychopathology is evident after 6 months, and longer-term follow-ups are required to gain accurate estimates of the longer-term psychological impacts from major natural disasters such as the 2016 wildfire.

In our study, the prevalence for likely problematic/hazardous drinking and drug use were 15% and 9.1%, respectively, at 18 months compared to rates of 10.3 and 14.0%, respectively, in the general population of Fort McMurray 6 months after the wildfire (34–36), suggesting a higher use of alcohol but a lower use of drugs in the period between 6 and 18 months after the wildfire.

The prevalence rate estimates for PTSD, MDD, and GAD in our sample are higher than the prevalence rate estimates for the general population in Canada. Thus, according to the 2012 Canadian Community Health Survey (44), 2.5% of Canadians aged 15 years and older reported symptoms compatible with GAD in the previous 12 months, and 5% of Canadians reported lifetime GAD. Similarly, the estimate 1 month and lifetime prevalence rates for PTSD among the general Canadian population are 2.4% and 9.2%, respectively (45). For major depression, 3.9% of Canadians aged 15 years and over reported symptoms that met the criteria for MDD in the previous 12 months, while 4.7% reported lifetime presence of MDD. Furthermore, Statistics Canada (46)reported 6.4% of Canadians meet the criteria for alcohol abuse or dependence annually with 4% meeting the criteria for drug abuse or dependence (46). All of these demonstrate the greatly increased rates of multiple psychiatric disorders following the wildfire when compared to the general Canadian population.

In our study, a history of anxiety disorders and receiving counseling after wildfire independently predicted higher likelihood of respondents developing PTSD. These two factors also independently predicted a higher likelihood for residents in the general Fort McMurray population to develop PTSD 6 months after the wildfires (36). Other studies found a pre-trauma history of mental disorders, especially mood and anxiety disorders, and conduct disorder as risk factors for developing PTSD (47–50). It is expected that people who are more severely impacted by the wildfires are more likely to seek and receive counseling, which might explain the association between receiving counseling and the likelihood of having a PTSD. Thus, it is unlikely there is any causal relationship between receiving counseling and developing PTSD. In contrast, receiving low level support from family and friends did not independently predict the likelihood for respondents in our sample to develop PTSD at 18 months as it did for the general population 6 months after the wildfire (36).

We identified four independent factors associated with development of likely MDD, namely: age, witnessed burning of homes, history of depressive disorder, and support from family/friends. Only one of these factors (support from family/friends) also predicted likely MDD in the general Fort McMurray population 6 months after the wildfire (34). In contrast, a history of anxiety disorder rather than a history of depressive disorder predicted the likelihood for respondents in the general population to develop MDD 6 months after the wildfire. Numerous studies have shown the importance of lack of social support as an independent predictor for depression after disasters (29, 51). Pre-disaster mental health status has also been emphasized as a significant risk factor for increasing the likelihood for victims to develop depression (52–55).

It may have been predicted that respondents who witnessed their property burnt had higher rates of subsequent MDD, but we found there was more than twice the likelihood of developing MDD in our study. Possible reasons for this could be greater incident exposure and/or experiencing an extra socio-economic stressor on top of other stressors (54, 56, 57). Our study suggests that respondents who are 40 years or less were twice as likely to meet the criteria for an MDD compared to those who were older than 40 years 18 months after the disaster. This is in contrast to the literature, which suggests that older persons have a greater likelihood of having depression (58) and might be more vulnerable in disasters (52) as they may have a greater likelihood of suffering physical illnesses that affect their mental health (59). Besides, middle aged households might perceive themselves as the main persons responsible for providing the necessities of their own families after a disaster, which could put more pressure and stress on them (59–61) and as such make them more vulnerable to developing depression. The contrast of the literature to our study could be due to the fact that most of the respondents who were over 40 years of age in our study were not elderly, with only 10 (3.4%) respondents being over 60 years old.

Our study suggests that only three variables were independently associated with likely GAD in our study sample, namely: being fearful for own lives or the lives of friends/family, history of depressive disorder, and history of anxiety disorder. This is in contrast to the six independent factors (witnessing of homes burning, place of residence after the wildfire, preexisting anxiety disorder, perceived support from the government or family/friends, post-crisis counseling), which predicted likely GAD in the general population 6 months after the wildfires (35). Preexisting mental health issues as a predictor of GAD after a disaster are one of the most replicated findings in many studies (62–66). The implicated reason for this finding could be the susceptibility of the people with mental health deficit to stress. It is possible that they have suboptimal reserve and coping skills necessary for dealing with disasters. On the other hand, “being fearful for own lives or the lives of friends/family” could be an indicator of the severity of the exposure, which has been highlighted in other studies as a risk factor for developing anxiety after disasters (67–72).

The reciprocal connection between the mental health problems and substance abuse has been well-documented previously, with around 47% of those with substance abuse problem having mental health problems and about 29% of those with a mental health disorder having a substance use disorder. Substance use disorder is especially common in people diagnosed with mood disorders, anxiety, and PTSD (73, 74). In addition, it has been shown in many studies that exposure to disasters can affect the alcohol and substance use (74–76). A few theories have been postulated to explain the connection between substance use and psychological distress after a disaster (77). One is that “disaster exposure decreases perceived coping self-efficacy, which, in turn, increases psychological distress and subsequently increases perceptions of self-medication in vulnerable individuals. These mechanisms lead to an increase in post-disaster substance use” (77).

Respondents with likely PTSD and likely depression but not likely GAD significantly self-reported increase alcohol use. However, when the AUDIT tool was used to assess their level of drinking, there was no statistically significant differences between those who had likely PTSD and MDD and those who meet the criteria for high risk, harmful, or hazardous drinking or alcohol dependence. It is possible that affected individuals increased their alcohol consumption from their own previous level although not heavily to be considered as “high risk, harmful or hazardous drinking or alcohol dependence.” Many other studies that gathered their information through self-report also showed increased alcohol consumption (75–79). On the contrary, our study found significant associations between problematic drug use and likely PTSD, MDD, and GAD as measured with the DUDIT tool.

To help address the major population mental health and addiction issues identified in our study, new approaches may be required. For example the possible utilization of innovative and cost-effective interventions such as supportive text messaging (80–85), and the rapid identification and referral of those most affected to secondary care, could help mitigate the mental health effects of the wildfires. Further studies are needed to explore the impact of population-based mental health interventions on the long-term mental health effects of the wildfires.

## Limitations of This Study

Because of resource constraints, we relied on self-report data from respondents. Under these conditions, no formal diagnosis was possible and so our analysis is based only on *likely* GAD, MDD, and PTSD diagnosis. Again, the study did not explore if the study participants had faced any other situations that may also have caused these disorders or aggravated them such as deaths, loss, or medical conditions, and therefore, a direct causal relationship of these disorders with the wildfires cannot be established. Furthermore, the sample of respondents was not fully representative of patients assessing all primary care services in Fort McMurray as only patients accessing the out-of-hours clinic that serves the entire city were surveyed. These limitations notwithstanding, the out-of-hours clinic is the only one serving the entire city and records in excess of 1,800 adult attendees monthly, thus providing the only opportunity to survey patients who access primary care services in Fort McMurray out-of-hours. Our study is therefore representative of the adult residents in Fort McMurray who access out-of-hours primary care services, and future studies could compare the prevalence rates of those accessing primary care services with the general population of Fort McMurray who are not accessing primary care services.

## Conclusions

Our study has measured prevalence rates for a wide variety of psychiatric conditions some 18 months after the Fort McMurray wildfire among residents seeking care from an out-of-hours primary care medical clinic. These include rates for PTSD, MDD, GAD, substance use, and alcohol use related problems. Our findings that there are significantly increased prevalence rates for these mental health and addiction conditions in this population support previous evidence that such natural disasters can have major long-term impacts on the mental health of survivors. They also suggest the need for enhanced or targeted screening and innovative treatments for these disorders at the primary care level after natural disasters.

## Data Availability Statement

The raw data supporting the conclusions of this manuscript will be made available by the authors, without reservations to any qualified researcher, subject to appropriate ethics or legal requirements.

## Ethics Statement

This study was carried out in accordance with the recommendations of the University of Alberta Review and Ethics Board. All subjects gave written informed consent in accordance with the Declaration of Helsinki. The protocol was approved by the University of Alberta Review and Ethics Board (Pro00066054).

## Author Contributions

VA conceived and designed the study, supervised data collection, analyzed the data, and jointly drafted the initial manuscript with SM. SM contributed to the study design and data imputation and jointly drafted the initial manuscript with VA. BN, IA, and SC participated in data collection, reviewing, and editing the initial draft of the manuscript. PC, AG, X-ML, and PS all contributed to data interpretation and editing the initial draft of the manuscript. All authors approved of the final draft of the manuscript before submission.

## Funding

The study was funded by the Department of Psychiatry, Faculty of Medicine, University of Alberta.

## Conflict of Interest

The authors declare that the research was conducted in the absence of any commercial or financial relationships that could be construed as a potential conflict of interest.
